# Quantitative Trait Locus Analysis of Late Leaf Spot Resistance and Plant-Type-Related Traits in Cultivated Peanut (*Arachis hypogaea* L.) under Multi-Environments

**DOI:** 10.1371/journal.pone.0166873

**Published:** 2016-11-21

**Authors:** Xiaojing Zhou, Youlin Xia, Junhua Liao, Kede Liu, Qiang Li, Yang Dong, Xiaoping Ren, Yuning Chen, Li Huang, Boshou Liao, Yong Lei, Liying Yan, Huifang Jiang

**Affiliations:** 1 Key Laboratory of Biology and Genetic Improvement of Oil Crops, Ministry of Agriculture, Oil Crops Research Institute of the Chinese Academy of Agricultural Sciences, Wuhan, Hubei, China; 2 Nanchong Academy of Agricultural Sciences, Nanchong, Sichuan, China; 3 National Key Laboratory of Crop Genetic Improvement, Huazhong Agricultural University, Wuhan, Hubei, China; 4 Department of Plant Science, University of Saskatchewan, Saskatoon, Saskatchewan, Canada; Mahatma Phule Krishi Vidyapeeth College of Agriculture, INDIA

## Abstract

Late leaf spot (LLS) is one of the most serious foliar diseases affecting peanut worldwide leading to huge yield loss. To understand the genetic basis of LLS and assist breeding in the future, we conducted quantitative trait locus (QTL) analysis for LLS and three plant-type-related traits including height of main stem (HMS), length of the longest branch (LLB) and total number of branches (TNB). Significant negative correlations were observed between LLS and the plant-type-related traits in multi-environments of a RIL population from the cross Zhonghua 5 and ICGV 86699. A total of 20 QTLs were identified for LLS, of which two QTLs were identified in multi-environments and six QTLs with phenotypic variation explained (PVE) more than 10%. Ten, seven, fifteen QTLs were identified for HMS, LLB and TNB, respectively. Of these, one, one, two consensus QTLs and three, two, three major QTLs were detected for HMS, LLB and TNB, respectively. Of all 52 unconditional QTLs for LLS and plant-type-related traits, 10 QTLs were clustered in five genetic regions, of which three clusters including five robust major QTLs overlapped between LLS and one of the plant-type-related traits, providing evidence that the correlation could be genetically constrained. On the other hand, conditional mapping revealed different numbers and different extent of additive effects of QTLs for LLS conditioned on three plant-type-related traits (HMS, LLB and TNB), which improved our understanding of interrelationship between LLS and plant-type-related traits at the QTL level. Furthermore, two QTLs, *qLLSB6-7* and *qLLSB1* for LLS resistance, were identified residing in two clusters of NB-LRR—encoding genes. This study not only provided new favorable QTLs for fine-mapping, but also suggested that the relationship between LLS and plant-type-related traits of HMS, LLB and TNB should be considered while breeding for improved LLS resistance in peanut.

## Introduction

Peanut or groundnut (*Arachis hypogaea* L.) is an important source of edible plant oil and protein and is cultivated in more than 100 countries. The total global production was 42.4 million tonnes and average yield was 1,653 kg ha^-1^ in 2014 (FAO, 2016) [1]. There is always a big gap between genetic potential of modern cultivars and their actual yield in the field. Yield losses are generally substantial when the crop is attacked by peanut diseases [2,3]. Late leaf spot (LLS) is one of the most serious foliar diseases caused by fungi [*Phaeoisariopsis personata*(Berk & M.A. Curtis)Arx] affecting peanut worldwide [4,5]. Peanut plants infected with this disease exhibit defoliating nature and result in over 50% yield loss [6]. Traditionally, LLS disease is controlled by fungicides, which are costly and toxic to the environment, therefore, the development of host-plant resistant cultivars has been considered as the primary strategy for controlling LLS disease.

Many important traits in agriculture are complex genetic behavior [7–9]. Genetic studies on LLS resistance suggest that resistance to this fungal disease is complex and polygenic in nature and sensitive to environments [10–15]. It has been long known that environment factors, such as temperature and humidity, are important affecting infection and development of *Phaeoisariopsis personata* [16–18]. In the field, a microclimate in which LLS may develop was directly influenced by plant-type-related traits. Several studies indicated associations between disease resistance and plant-type-related traits [19–21]. However, the genetic basis of LLS and relationship between LLS and plant-type-related traits in peanut are poorly understood.

QTL mapping is essential for identifying the genomic regions that control traits of interest before deploying linked markers in the breeding using marker-assisted selection (MAS) and also facilitate gene mining [22–25]. In recent years, a number of QTLs for LLS resistance have been identified in peanut. Khedikar et al. (2010) identified 11 minor QTLs for LLS using a partial linkage map comprising 56 SSR loci [14]. Sujay et al. (2012) identified 13 QTLs including five major QTLs for LLS in TAG 24 × GPBD 4 (RIL-4) population, and 15 QTLs including eight major QTLs for LLS in TG 26 × GPBD 4 (RIL-5) population, using the linkage maps with 188 and 181 loci in RIL-4 and RIL-5, respectively [15]. Based on a consensus map of these two populations, two candidate genomic regions conferring resistance to LLS were reported [15]. In the case of plant-type-related traits of height of the main stem (HMS), length of the longest branch (LLB) and total number of branches (TNB), only a few QTLs have been identified so far. Huang et al. (2014) identified three minor QTLs for HMS and two minor QTLs for TNB by using a genetic linkage map containing 470 simple sequence repeat (SSR) loci for cultivated peanut [26]. Shirasawa et al. (2012) identified three QTLs for HMS; two QTLs for LLB and one QTL for TNB with a genetic map containing 1,114 loci [27]. A major limitation of these studies was that the analyses were based on linkage maps mainly using simple sequence repeat (SSR) markers in which many regions were sparsely represented, thus it was not possible to obtain precise information about the numbers and locations of the QTLs. On the other hand, although the genetic linkage maps based on abundant single nucleotide polymorphism (SNP) markers could greatly increase marker density, there were rare cases of completeness and the maps have not been applied in QTL research in peanut yet [28,29].

Our previous study reported development of a SNP-based genetic linkage map consisting of 1,685 loci using the RIL population of Zhonghua 5 × ICGV 86699 [29]. In present study, we conducted QTL identification for resistance to LLS and the plant-type-related traits of HMS, LLB and TNB in multi-environments using this linkage map to investigate the genetic basis of LLS and the interrelationship between LLS and the plant-type-related traits.

## Materials and Methods

### Ethics statement

This article does not contain any studies with human participants or animals performed by any of the authors.

### Plant material and field design

A RIL population including 166 lines derived from a cross between maternal parent of Zhonghua 5 and paternal parent of ICGV 86699 was employed in this study for mapping and trait analysis. Zhonghua 5 is a popular cultivar in China that is susceptible to LLS [29,30]. ICGV 86699 is a breeding variety which is highly resistant to LLS. ICGV 86699 has wild-derived source of LLS resistance and its pedigree is [*Arachis batizocoi*/*A*. *duranensis*//*A*. *hypogaea* cv. NC 2]—CS 29 [31]. In addition, the two parents have significant differences in HMS, LLB, TNB and other traits such as insect resistance and other disease resistances [31].

The RIL lines were grown using a randomized complete block design with two replications at two experimental stations. One is located in the experimental farm at Oil Crops Research Institute (OCRI) of Chinese Academy of Agricultural Sciences (CAAS), Wuhan (WH), Hubei, China (30°35’N, 114°33’E). The other experimental station is located at Nanchong Academy of Agricultural Sciences, Nanchong (NC), Sichuan, China (30°80’N, 106°06’E). The seeds were sown at the end of April of each experimental year. Each accession was planted in a single row, with 8–10 plants in each row and 10 cm intervals between plants within each row and 30 cm intervals between rows. The parental genotypes were also sown after every 50 rows as controls.

### Phenotypic observation

The parents and 166 RIL lines were used to assess LLS resistance and plant-type-related traits of HMS, LLB and TNB. For LLS, phenotyping was carried out in NC in year 2011 (NC11), year 2012 (NC12), year 2013 (NC13), and in WH in year 2012 (WH12) and year 2013 (WH13). The traits of HMS, TNB and LLB were investigated in two consecutive years (2012 and 2013) in NC and WH. Each location and year combination was considered as individual experimental environment.

The investigation of LLS in NC was under natural environment as this region belongs to the special basin terrain and is easy for the occurrence and epidemic of LLS disease. The average lowest ~ highest temperature, average day length, and average humidity of experiment periods of 2011–2013 in NC presented as following: 22.4~30.2°C/138.9h/88% (June, 2011), 24.3~31.6°C/185.4h/86% (July, 2011), 25.8~34.3°C/197.0h /83% (August, 2011); 21.6~28.5°C/129.7h/85% (June, 2012), 24.3~31.1°C/136.9h/83% (July, 2012), 25.3~33.7°C/183.0/82% (August, 2012); 23.3~32.1°C/135.5h/80% (June, 2013), 26.1~33.6°C/187.3h/79% (July, 2013), 25.6~33.5°C/191.3h/80% (August, 2013). While LLS in WH was created using artificial disease epiphytotic since the region is not conducive to the epidemic of LLS disease. Methods for isolating LLS conidia and inoculating field plants were conducted as described in Khedikar et al. (2010) [14]. The average lowest ~ highest temperature, average day length, and average humidity of experiment periods of 2012–2013 in WH were as following: 22.8~31.4°C/192h/81% (June, 2012), 26.9~34.4°C/196h/77% (July, 2012), 25.1~32.3°C/198h/69% (August, 2012); 22.5~30.8°C/200.5h/81% (June, 2013), 27.4~34.6°C/287.5h/72% (July, 2013), 26.9~35.1°C/ 281.7h/69% (August, 2013). After 120 days of sowing, severity of LLS was assessed using a 1 to 9 severity scale in both places [32].

Height of main stem, length of the longest branch and total number of branches were measured at the harvesting stage (after 130 days of sowing). The unit of measured values of HMS and LLB was centimeter (cm). TNB was counted by the number of branches. A schematic diagram of HMS and LLB was shown in S1 Fig.

### Statistical analysis

The broad-sense heritability was calculated according to Hallauer and Miranda (1998) [33] as *h*^2^ = σ^2^_g_/(σ^2^_g_+ σ^2^_ge_ / n + σ^2^_e_ / nr), where σ^2^_g_ was the genetic variance, σ^2^_ge_ was the interaction variance of the genotype with environment, σ^2^_e_ was the variance of residual error, n was the number of environments and r was the number of replications. The estimation of the variance components was obtained using the SAS software by general linear model (GLM) procedure [34]. Correlation coefficients between traits across environments were estimated using the PROC CORR procedure of SAS.

### Unconditional QTL mapping

Detection of QTL in each environment was performed by using the composite interval mapping (CIM) program of the Windows QTL Cartographer 2.5 [35]. The forward regression method model 6 (default model) was selected to obtain covariates. The number of control markers, window size and walk space were set to 5, 10 and 2 cM, respectively. A LOD threshold of 2.5 was chosen to declare a putative QTL as significant [36]. QTLs repeatedly identified with clearly similar positions (overlapping 1-LOD confidence intervals) for the same trait in different environments were integrated into a consensus QTL [37]. QTLs detected for different traits with overlapped confidence intervals and common marker(s), or couples of overlapped QTLs with distance less than 2 cM was defined as a QTL cluster in which at least one major QTL with PVE >10% was included [37]. Average values of two replicates for each trait in a single environment were used as phenotypic data for single environment QTL analysis.

Joint QTL was analyzed by mixed linear model-based composite interval mapping (MCIM) of QTLNetwork software version 2.0 [38]. Phenotypic values of each trait in all environments were averaged for joint QTL analysis. The LOD thresholds were determined by a 1,000 permutation test at a 95% confidence level. The nomenclature of QTL was similar to that described by McCouch et al. (1997) [39]. QTL was designated with initial letter “q” followed by trait name, linkage groups and serial number (if more than one QTL of the same trait exist in the same chromosome), respectively.

### Conditional QTL mapping

The phenotypic values for conditional QTL analysis was showed as y (T1|T2), where T1|T2 indicated that trait 1 conditioned on trait 2. y (T1|T2) was obtained by the mixed model approach using software QGAStation 2.0 (http://ibi.zju.edu.cn/software/qga/index.htm) [40,41]. In this study, the correlation analysis based on the phenotypic data showed significant negative correlation between LLS and plant-type-related traits. The QTL-clusters showed that the QTLs controlled different traits were mapped on the same genomic region. To further investigate the genetic interrelationship between LLS resistance and plant-type-related traits,we performed conditional QTL mapping with correlated traits as proposed by Zhao et al. (2006) [42]. Conditional phenotypic values including LLS|HMS, LLS|LLB and LLS|TNB in the four environments (NC12, NC13, WH12 and WH13) were calculated. Conditional QTL mapping was performed by the composite interval mapping method as described above for unconditional QTLs.

## Results

### Trait performance

**Fig 1:**
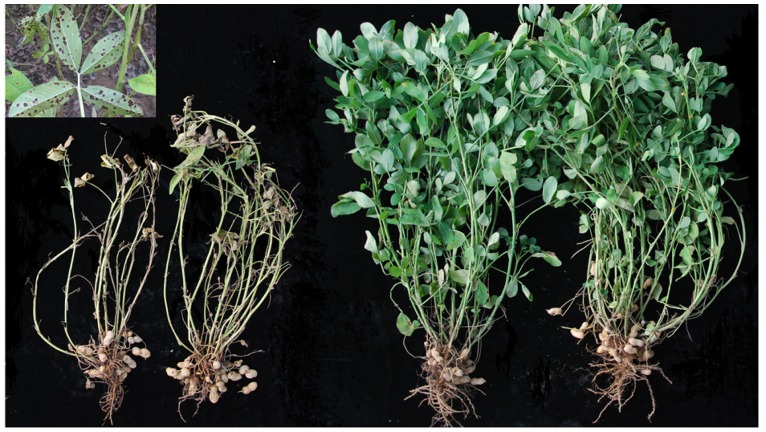
Phenotypes of two parental inbred lines used for QTL mapping in this study. Plants were photographed at 120 days after sown on soil. Left two lines were Zhonghua 5, right two lines were ICGV 86699. The picture of top left corner showed infected leaves by late leaf spot.

The two parents, Zhonghua 5 and ICGV 86699, showed significant differences (P < 0.001) for LLS and plant-type-related traits of HMS, LLB and TNB. ICGV 86699 consistently exhibited lower disease score of LLS and higher values of the three plant-type-related traits than Zhonghua 5 (Fig 1, Table 1). The RIL individuals showed a continuous distribution for all traits across different environments (S2 Fig), which suggested a quantitative inheritance pattern for the investigated traits.

**Table 1:** Phenotypic performance of the four traits in parents and RIL lines under multi-environments.

Trait	Env.	Zhonghua 5	ICGV 86699	Population					
				Mean	SD	Min	Max	Skew	Kurt
LLS	NC11	8.3	1.0	5.1	1.8	1.0	9.0	0.01	-0.81
	NC12	9.0	2.7	5.3	1.9	1.3	9.0	0.41	-0.78
	NC13	8.0	1.3	5.8	1.9	1.3	9.0	-0.42	-0.66
	WH12	8.1	2.3	5.3	2.0	1.7	9.0	-0.06	-0.94
	WH13	8.0	2.7	5.0	2.5	1.0	9.0	0.08	-0.89
HMS	NC12	27.3	51.6	38.9	8.1	21.2	68.4	0.69	0.96
	NC13	50.4	82.3	62.6	11.2	36.8	105.7	0.98	1.39
	WH12	47.2	80.4	56.9	12.0	23.8	89.6	0.11	-0.55
	WH13	42.3	72.2	59.7	13.6	35.0	97.9	0.26	-0.71
LLB	NC12	46.3	70.1	53.6	9.7	35.8	90.4	0.77	0.87
	NC13	56.2	94.3	73.9	10.7	48.5	106.7	0.6	0.11
	WH12	42.2	91.2	65.3	13.9	35.0	102.0	0.05	-0.58
	WH13	50.4	80.4	69.6	15.5	41.5	112.8	0.33	-0.61
TNB	NC12	11.6	45.6	18.7	9.1	8.2	51.8	0.98	0.96
	NC13	13.2	33.6	18.7	6.5	8.7	45.4	0.80	0.97
	WH12	10.0	28.2	18.0	6.6	6.8	42.7	0.98	0.99
	WH13	11.5	29.4	18.2	9.9	6.0	55.7	1.05	1.12

Zhonghua 5: maternal parent; ICGV86699: paternal parent.

LLS (late leaf spot disease) was assessed by 1–9 scale disease score; HMS (height of main stem) and LLB (length of the longest branch) were measured in the unit of centimeter (cm); TNB (total number of branches) was counted by the number of branches.

Env. represents environment; NC11, NC12, NC13, WH12 and WH13 represent Nanchong in 2011, 2012, 2013respectively; WH12 and WH13 represent Wuhan in 2012 and 2013, respectively.

SD: standard deviation.

ANOVA of all four traits across environments indicated that genotype (G), environment (E) and genotype-environment interactions (G×E) had significant effects on the traits. The four traits (LLS, HMS, LLB and TNB) showed high broad-sense heritability of 86.9%, 75.0%, 77.5% and 82.2%, respectively (Table 2), which suggested that genetic factors played a major role in the expression of these traits with significant environmental variation affecting these traits. To pinpoint their relationships, correlation coefficients (*r*) between the traits across environments were calculated (Table 3). The disease score of LLS of the RIL population exhibited significant (P < 0.05) negative correlations with three plant-type-related traits in all environmental cases, except in NC13 which had no significant phenotypic correlation between LLS and HMS. Significant positive relationships have been found between different plant-type-related traits, especially for HMS and LLB which showed the strongest correlation.

**Table 2:** Variance (ANOVA) Analysis and the broad-sense heritability (*h*^*2*^) for the four traits of RIL lines in multi-environments.

Trait	Source	DF	Sum of square	Mean square	*F* value	*P*	*h*^2^
LLS	Genotype	165	4155.82	25.19	49.3	< .0001	86.86
	Environment	4	180.05	45.01	88.1	< .0001	
	Genotype×Environment	654	2164.65	3.31	6.48	< .0001	
	Error	1154	589.62	0.51			
HMS	Genotype	165	133791.44	810.86	22.05	< .0001	74.95
	Environment	3	73007.51	24335.84	661.87	< .0001	
	Genotype×Environment	493	100121.08	203.09	5.52	< .0001	
	Error	982	36106.39	36.77			
LLB	Genotype	165	172190.66	1043.58	17.56	< .0001	77.5
	Environment	3	56379.56	18793.19	316.2	< .0001	
	Genotype×Environment	493	115751.2	234.79	3.95	< .0001	
	Error	983	58424.8	59.46			
TNB	Genotype	165	66507.18	403.07	18.31	< .0001	82.2
	Environment	3	253.55	84.52	21.84	< .0001	
	Genotype×Environment	492	35278.45	71.7	3.26	< .0001	
	Error	974		22.01			

LLS: late leaf spot disease; HMS: height of main stem; LLB: length of the longest branch; TNB: total number of branches.

DF indicates degree freedom.

*h*^*2*^ indicates the broad-sense heritability.

**Table 3:** Correlation analysis for the four traits across multi-environments.

Trait	Env.	LLS	HMS	LLB
HMS	NC12	-0.22**		
	NC13	-0.14^ns^		
	WH12	-0.56**		
	WH13	-0.19*		
LLB	NC12	-0.31**	0.84**	
	NC13	-0.19*	0.9**	
	WH12	-0.58**	0.96**	
	WH13	-0.21**	0.94**	
TNB	NC12	-0.55**	0.24**	0.42**
	NC13	-0.47**	0.23**	0.15*
	WH12	-0.22**	0.21**	0.23**
	WH13	-0.23**	0.24**	0.28**

Env. represents environments; NC12 and NC13 represent Nanchong in 2012 and 2013 respectively; WH12 and WH13 represent Wuhan in 2012 and 2013, respectively.

*, ** and ns indicate significance at *P* < 0.05, *P* < 0.01 and non-significant effect, respectively.

### QTL analysis for LLS and plant-type-related traits

To identify QTLs for LLS, HMS, LLB and TNB, phenotypic data were analyzed together with genetic mapping data of 1,685 markers [29] (S1 Table). A total of 52 significant QTLs were identified for all four traits through single-environment QTL analysis (Table 4, S3 Fig). The QTLs were distributed on 15 chromosomes and explained 3.41% to 19.12% phenotypic variation. Six QTLs (*qLLSA1-3*, *qLLSB6-2*, *qHMSA9-2*, *qLLBA9-2*, *qTNBA8* and *qTNBB6-3*) were identified in multiple environments. Joint QTL analysis detected 18 putative QTLs associated with the four traits (Table 4, S2 Table) and 12 of these QTLs were coincident with the results of single-environment mapping (Table 4).

**Table 4:** QTLs identified for LLS and plant-type-related traits through single-environment QTL mapping using high density SNP-based map.

Trait	QTL^a^	Chr.^b^	Marker interval	Range (cM)	Env.^c^	Position	Additive^d^	PVE^e^ (%)	LOD
LLS	*qLLSA1-1*	A1	Ahsnp978-Ahsnp275	30.3–33.5	NC13	32.01	0.46	5.12	3.47
	*qLLSA1-2*	A1	Ahsnp517-Ahsnp422	38–43.4	NC13	39.11	0.48	5.63	3.89
	*qLLSA1-3*	A1	Ahsnp1339-Ahsnp819	45.3–52	WH13	46.51	0.64	5.07	2.58
					NC13	48.51	0.49	5.11	3.32
	*qLLSA4-1*	A4	Ahsnp1681-Ahsnp1897	50.4–52.7	NC13	51.51	0.52	5.29	3.66
	*qLLSA5*	A5	Ahsnp1180-Ahsnp78	20.6–28.1	NC13	25.81	0.38	3.64	2.56
	qLLSA6-1*	A6	Ahsnp629-Ahsnp1883	11.4–14.6	NC12	13.71	0.39	3.41	2.85
	*qLLSA6-2*	A6	Ahsnp1843-Ahsnp1521	19.2–24.2	WH12	21.61	0.47	4.31	2.57
	*qLLSA6-3*	A6	Ahsnp419-Ahsnp418	31–35.8	WH12	33.61	0.67	7.6	3.96
	*qLLSA8*	A8	Ahsnp320-Ahsnp919	57–76.1	NC12	66.41	0.39	3.69	3.09
	*qLLSA10*	A10	Ahsnp1559-Ahsnp809	27.5–31.1	NC11	31.01	0.69	10.26	6.37
	qLLSB1*	B1	GM1331-Ahsnp1805	0–8.8	WH12	4.41	0.64	8.22	5.06
	qLLSB4-1*	B4	Ahsnp505-Ahsnp471	11.4–32.9	NC11	22.54	0.52	5.75	3.38
	qLLSB6-1*	B6	Ahsnp295-AHGS1422	31.8–40.1	NC11	35.36	0.74	13.89	8.17
	*qLLSB6-2*	B6	Ahsnp539-Ahsnp136	44.5–47	NC12	45.81	0.75	11.66	8.92
					NC11	45.81	0.74	12.9	7.81
	*qLLSB6-3*	B6	Ahsnp1070-Ahsnp1665	56–57.6	NC12	57.01	0.6	7.16	4.23
	*qLLSB6-4*	B6	Ahsnp676-Ahsnp231	62.18–64.62	NC13	63.61	0.89	18.47	10.79
	*qLLSB6-5*	B6	Ahsnp231-AHGS1431	64.9–67.8	WH13	65.31	0.69	6.33	2.9
	*qLLSB6-6*	B6	AHGS1431-Ahsnp163	68.4–71.5	NC13	71.01	0.91	19.12	11.25
	*qLLSB6-7*	B6	Ahsnp1166-Ahsnp996	73.3–78	WH13	74.51	1.03	14.18	6.85
	*qLLSB10*	B10	Ahsnp1458-Ahsnp299	5.3–8.6	NC11	7.51	0.44	4.1	2.73
HMS	*qHMSA5-1*	A5	Ahsnp1180-Ahsnp1133	21.6–25.7	NC13	23.71	-5.64	15.28	4.88
	*qHMSA5-2*	A5	Ahsnp1338-Ahsnp213	33.6–38	NC13	35.61	-4.81	10.66	3.46
	*qHMSA9-1*	A9	Ahsnp1103-Ahsnp1059	0–2.2	NC13	0.01	-3.91	10.73	4.98
	qHMSA9-2*	A9	Ahsnp902-Ahsnp1167	71.6–82.2	WH12	73.11	-3.08	5.39	3.06
					WH13	75.11	-3.36	5.22	2.68
	*qHMSA10-1*	A10	Ahsnp77-Ahsnp787	7.8–8.8	WH12	7.91	-3.22	5.26	2.98
	*qHMSA10-2*	A10	Ahsnp712-GM692	14.4–19.9	WH12	17.81	-3.31	6.14	3.49
	*qHMSB2*	B2	Ahsnp1331-Ahsnp1308	1.6–12.1	NC13	5.01	3.97	8.94	4.24
	*qHMSB3-1*	B3	Ahsnp586-Ahsnp1194	48–52.2	WH13	50.21	-3.39	5.39	2.93
	qHMSB3-2*	B3	Ahsnp1236-Ahsnp1549	59.7–61.8	WH13	60.81	-4.02	7.99	4.63
	qHMSB6*	B6	Ahsnp227-Ahsnp295	12.1–32.3	WH13	24.11	-3.22	4.76	2.78
LLB	*qLLBA9-1*	A9	Ahsnp1103-Ahsnp1059	0–2.4	NC13	0.01	-3.7	10.59	5.09
	*qLLBA9-2*	A9	Ahsnp902-Ahsnp1167	72.2–82.2	WH13	77.11	-4.05	5.55	2.75
					WH12	77.11	-5	11.45	5.8
	*qLLBB1*	B1	Ahsnp966-Ahsnp1373	28.2–31.1	WH12	30.91	-3.61	5.17	3.02
	*qLLBB3*	B3	Ahsnp1236-Ahsnp1549	59.3–61.8	WH13	60.81	-3.61	4.73	2.68
	qLLBB6*	B6	Ahsnp227-Ahsnp295	16.6–31.1	WH12	27.81	-2.93	4	2.55
	*qLLBB8-1*	B8	AHGS1388-Ahsnp1644	11.5–16	NC12	14.71	-2.86	6.86	3.25
	qLLBB8-2*	B8	Ahsnp779-Ahsnp732	62.6–73.4	WH12	66.31	-3.25	4.28	2.71
TNB	*qTNBA3*	A3	Ahsnp965-Ahsnp1014	35.7–39.2	WH12	36.81	-1.78	6.19	3.25
	*qTNBA5*	A5	Ahsnp926-Ahsnp236	15.7–17.8	WH12	17.51	-1.78	5.22	2.87
	*qTNBA6-1*	A6	Ahsnp418-AHGS0153	37.2–43.6	WH12	42.11	1.8	4.59	2.66
	*qTNBA6-2*	A6	Ahsnp19-Ahsnp1889	51.5–57.5	WH12	55.51	1.97	6.05	2.87
	qTNBA8*	A8	Ahsnp1305-Ahsnp1244	23.4–41.9	NC12	32.71	-2.32	5	2.76
					NC13	28.41	-1.63	5.34	3.06
					NC13	40.71	-1.6	4.96	2.7
	*qTNBA10*	A10	ARS775-Ahsnp1387	0–6.8	NC12	0.01	-2.61	6.96	3.81
	qTNBB1*	B1	GM1331-Ahsnp642	0–2	WH13	0.01	-2.67	5.58	3.3
	*qTNBB3*	B3	AHGS1201-AHGS2526	12.6–25.9	WH13	17.51	3.98	5.32	3.56
	*qTNBB4-1*	B4	Ahsnp781-Ahsnp1160	46.8–47.8	WH13	46.81	-2.88	5.76	3.4
	*qTNBB4-2*	B4	Ahsnp207-Ahsnp232	54.7–56.6	WH13	55.61	-3.29	9.54	5.82
	*qTNBB4-3*	B4	Ahsnp282-Ahsnp1066	64.9–68.3	WH13	67.81	-3.67	11.85	6.48
	*qTNBB6-1*	B6	Ahsnp425-Ahsnp349	48.9–62.2	NC13	54.51	-2.32	9.8523	4.77
	*qTNBB6-2*	B6	Ahsnp1309-Ahsnp64	62.6–65	WH13	63.61	-3.58	10.35	5.92
	qTNBB6-3*	B6	AHGS1431-Ahsnp690	69.5–71.5	NC13	71.01	-2.05	8.1	4.74
					WH13	71.01	-4.05	13.44	7.95
	*qTNBB8*	B8	Ahsnp1700-Ahsnp1055	10.1–14.2	WH12	10.81	-2.05	7.55	4.16

^a^ The nomination of QTL is comprised of four parts. The first part “q” stands for QTL. The second part is the abbreviation of traits. The third part, the number stands for chromosome. The fourth part is the serial number of QTL on the same chromosome. The names followed by * were QTLs simultaneously detected by joint QTL analysis among all environments.

^b^ Chr. indicates chromosome.

^c^ Env. indicates environment. NC11, NC12, NC13 WH12 and WH13 represent Nanchong in 2011, Nanchong in 2012, Nanchong in 2013, Wuhan in 2012 and Wuhan 2013, respectively.

^d^.Additive effect of the QTL.

^e^ The phenotypic variation explained of corresponding QTL.

#### Late leaf spot resistance

A total of 20 QTLs controlling LLS were identified in all investigated environments (Table 4, S3 Fig) and were mapped on chromosomes A1, A4, A5, A6, A8, A10, B1, B4, B6 and B10. Chromosome B6 had seven QTLs, the most number of QTLs of all chromosomes. Phenotypic variation explained by individual QTL ranged from 3.41% to 19.12% and by QTL together in single environment ranged from 25.58% to 62.38%. Among them, six QTLs (*qLLSA10*, *qLLSB6-1*, *qLLSB6-2*, *qLLSB6-4*, *qLLSB6-6* and *qLLSB6-7*) with over 10% PVE were considered as major QTLs. Two QTLs (*qLLSA1-3* and *qLLSB6-2*) were identified in multiple environments, including the major QTL *qLLSB6-2* which was accounted for 11.66–12.90% PVE with LOD value 7.81–8.92. In addition, LLS resistance alleles at all QTLs were contributed by ICGV 86699.

#### Height of main stem

For HMS, 10 QTLs distributed on six chromosomes (A5, A9, A10, B2, B3 and B6) were identified. They individually accounted for 4.76–15.28% of the phenotypic variation and totally explained 16.79–45.61% of the HMS variation in each environment (Table 4, S3 Fig). Three major QTLs (*qHMSA5-1*, *qHMSA5-2* and *qHMSA9-1*) were identified. The *qHMSA9-2* was a consensus QTL detected in WH12 and WH13. The *qHMSA9-2* together with two environment-specific QTLs (*qHMSB3-2* and *qHMSB6*) was also identified by joint analysis. The alleles for increasing HMS value at nine QTL regions were contributed by ICGV 86699, while the allele for increasing trait value at one QTL region was contributed by Zhonghua 5.

#### Length of the longest branch

Seven QTLs influencing LLB were identified on chromosomes A9, B1, B3, B6 and B8. They individually displayed 4.00% to 11.45% PVE and totally displayed 6.86–24.90% cumulative PVE for LLB in each environment (Table 4, S3 Fig). Two major QTLs (*qLLBA9-1* and *qLLBA9-2*) were identified. One of the major QTL *qLLBA9-2* explained 5.55% and 11.45% of the phenotypic variation in the WH12 and WH13 environments, respectively. Six QTLs were environment-specific. The alleles for increasing LLB value at all QTL regions were contributed by ICGV 86699.

#### Total number of branches

A total of 15 QTLs for TNB were identified on chromosomes A3, A5, A6, A8, A10, B1, B3, B4, B6 and B8 (Table 4, S3 Fig). They individually displayed 4.59–13.44% PVE with cumulative PVE of 11.96 to 61.84% for TNB across environments. Two QTLs (*qTNBA8* and *qTNBB6-3*) were identified across multi-environments, and they were also identified in correspondence with joint analysis. Three major QTLs, *qTNBB4-3*, *qTNBB6-2* and *qTNBB6-3* were identified, in which *qTNBB6-3* explained 8.10% and 13.44% of the phenotypic variation in NC13 and WH13, respectively. The alleles for increasing TNB value at twelve QTL regions were contributed by ICGV 86699, while the allele for increasing the trait value at three QTL regions were contributed by Zhonghua 5.

### Clusters with co-located QTLs

Five QTL clusters were identified on chromosomes A5, A9 and B6 (Table 5, S3 Fig). Among them, three clusters overlapped between LLS and one of plant-typed traits. Clusters IV and V, both located on chromosome B6, included QTL for LLS co-located with QTL for TNB. It is worth mentioning that four QTLs (*qLLSB6-4* and *qTNBB6-2*, *qLLSB6-6* and *qTNBB6-3*) in the two clusters were all displayed PVE >10%, in which one QTL (*qTNBB6-3*) was detected in multi-environments. QTL for LLS overlapped with QTL for HMS located on A5. These results indicated that obviously genetic correlation between LLS and plant-type-related traits. In addition, clusters II and III showed co-located QTLs for HMS and LLB on chromosomes A9.

**Table 5:** QTL clusters for four traits examined in this study.

QTL cluster No.	Chr.	Trait included	QTL	Marker interval	Range (cM)	Env.	Pos.	PVE (%)	A
I	A5	LLS+HMS	*qLLSA5*	Ahsnp1180-Ahsnp78	20.6–28.1	NC13	25.81	3.64	-0.38
			*qHMSA5-1*	Ahsnp1180-Ahsnp1133	21.6–25.7	NC13	23.71	15.28	5.64
II	A9	HMS+LLB	*qHMSA9-1*	Ahsnp1103-Ahsnp1059	0–2.2	NC13	0.01	10.73	3.91
			*qLLBA9-1*	Ahsnp1103-Ahsnp1059	0–2.4	NC13	0.01	10.59	3.70
III	A9	HMS+LLB	*qHMSA9-2*	Ahsnp902-Ahsnp1167	71.6–82.2	WH12	73.11	5.39	3.08
						WH13	75.11	5.22	3.36
			*qLLBA9-2*	Ahsnp902-Ahsnp1167	72.2–82.2	WH12	77.11	11.45	5
						WH13	77.11	5.55	4.05
IV	B6	LLS+TNB	*qLLSB6-4*	Ahsnp676-Ahsnp231	62.18–64.62	NC13	63.61	18.47	-0.89
			*qTNBB6-2*	Ahsnp1309-Ahsnp64	62.6–65	WH13	63.61	10.35	3.58
V	B6	LLS+TNB	*qLLSB6-6*	AHGS1431-Ahsnp163	68.4–71.5	NC13	71.01	19.12	-0.91
			*qTNBB6-3*	AHGS1431-Ahsnp690	69.5–71.5	NC13	71.01	8.1	2.05
						WH13	71.01	13.44	4.05

Chr. represents chromosome.

PVE indicates the percentage of phenotypic variation explained by corresponding QTL.

A represents the additive effect.

### Conditional analysis

Conditional analysis was performed to determine genetic relationship between LLS and plant-type-related traits. We identified 21 conditional QTLs for LLS distributed on chromosomes A1, A2, A3, A4, A6, B2, B3, B4 and B6 in four environments (NC12, NC13, WH12 and WH13). Of the 21 conditional QTLs, 10 were also detected as unconditional QTLs and 11 were detected as new QTLs (Table 6). Six unconditional QTLs were not recaptured under conditional analysis (Table 6).

**Table 6:** The unconditional and conditional QTLs for LLS in the four environments (NC12, NC13, WH12, WH13).

QTL	Marker interval	LLS			LLS|HMS			LLS|LLB			LLS|TNB		
		Env.	A	PVE	Env.	A	PVE	Env.	A	PVE	Env.	A	PVE
*qLLSA1-1*	Ahsnp978-Ahsnp275	NC13	0.46	5.12	NC13	0.47	6.12	NC13	0.55	8.05			
*qLLSA1-2*	Ahsnp517-Ahsnp422	NC13	0.48	5.63	NC13	0.5	6.91	NC13	0.63	9.99	NC13	0.48	6.24
					NC12	0.46	5.67	NC12	0.4	4.57			
*qLLSA1-3*	Ahsnp1339-Ahsnp819	NC13	0.49	5.11	NC13	0.45	4.99	NC13	0.57	7.21	NC13	0.63	9.46
		WH13	0.64	5.07	NC12	0.45	4.98				NC12	0.46	5.93
*qLLSA1-4*	Ahsnp89-Ahsnp1913										WH13	0.8	7.7
*qLLSA2*	AHGS1495-Ahsnp1397							WH13	0.82	7.52			
*qLLSA3*	Ahsnp1321-Ahsnp988				NC12	0.41	3.8				WH12	0.52	4.57
*qLLSA4-1*	Ahsnp1681-Ahsnp1897	NC13	0.52	5.29				NC13	0.51	6.05	NC13	0.56	7.92
*qLLSA4-2*	Ahsnp237-Ahsnp667							NC12	0.4	4.01			
*qLLSA5*	Ahsnp1180-Ahsnp78	NC13	0.38	3.64									
*qLLSA6-1*	Ahsnp629-Ahsnp1883	NC12	0.39	3.41									
*qLLSA6-2*	Ahsnp1843-Ahsnp1521	WH12	0.47	4.31	WH12	0.49	4.5				WH12	0.4	5.2
*qLLSA6-3*	Ahsnp419-Ahsnp418	WH12	0.67	7.6	WH12	0.67	8	WH12	0.65	7.8	WH12	0.68	8.0
*qLLSA6-4*	Ahsnp1731-Ahsnp1889										NC12	0.42	4.86
*qLLSA8*	Ahsnp320-Ahsnp919	NC12	0.39	3.69									
*qLLSB1*	GM1331-Ahsnp1805	WH12	0.64	8.22									
*qLLSB2-1*	Ahsnp1281-Ahsnp1308				WH13	1.01	12.34						
*qLLSB2-2*	Ahsnp784-Ahsnp1260				WH13	0.81	8.39						
*qLLSB2-3*	Ahsnp784-Ahsnp1694										WH13	0.77	8.4
*qLLSB3*	AHGS1201-AHGS1661										WH13	0.87	8.72
*qLLSB4-2*	Ahsnp108-Ahsnp789										WH12	0.54	6.29
*qLLSB4-3*	Ahsnp239-Ahsnp929										WH12	0.57	7.26
*qLLSB6-2*	Ahsnp539-Ahsnp136	NC12	0.75	11.66									
*qLLSB6-3*	Ahsnp1070-Ahsnp1665	NC12	0.6	7.16									
*qLLSB6-4*	Ahsnp676-Ahsnp231	NC13	0.89	18.47	NC13	0.71	11.87	NC13	0.85	19.41			
					NC12	0.7	11.32	NC12	0.67	10.85			
*qLLSB6-5*	Ahsnp231-AHGS1431	WH13	0.69	6.33	WH13	0.52	5.74	WH13	0.47	4.68			
*qLLSB6-6*	AHGS1431-Ahsnp163	NC13	0.91	19.12	NC13	0.76	13.89	NC13	0.92	22.55	NC13	0.44	5.42
					NC12	0.77	14.42	NC12	0.74	14.16	NC12	0.44	5.2
*qLLSB6-7*	Ahsnp1166-Ahsnp996	WH13	1.03	14.18	WH13	1.66	11.15	WH13	0.91	8.98			
					WH12	0.55	6.86	WH12	0.53	6.5			

A indicates the additive effect of the QTL.

PVE: percentage of phenotypic variance explained by the QTL.

LLS|HMS: late leaf spot conditioned on height of main stem.

LLS|LLB: late leaf spot conditioned on length of the longest branch.

LLS|TNB: late leaf spot conditioned on total number of branches.

Conditional QTL mapping of LLS|HMS revealed 12 QTLs. Nine marker intervals encompassed both unconditional and conditional QTLs, of which five (*qLLSA1-2*, *qLLSA1-3*, *qLLSB6-4*, *qLLSB6-6* and *qLLSB6-7*) were detected in multiple environments. Furthermore, the three QTLs (*qLLSB6-4*, *qLLSB6-6* and *qLLSB6-7*) were also detected with PVE over 10%. Of the nine QTLs detected in both strategies, five conditional QTLs changed additive effects significantly and four conditional QTLs showed similar additive effects compared to the corresponding unconditional QTLs. Three new QTLs for LLS|HMS were detected and seven unconditional QTLs were not identified again.

Conditional QTL mapping of LLS|LLB identified 11 QTLs. Nine were detected with the same marker intervals as unconditional QTLs, and four of them (*qLLSA1-2*, *qLLSB6-4*, *qLLSB6-6* and *qLLSB6-7*) were detected in multiple environments. When LLS was conditioned on LLB, *qLLSB6-4* displayed 10.85% and 19.41% PVE in NC12 and NC13, respectively. The *qLLSB6-6* was also identified at both NC12 and NC13 and displayed 14.16% and 22.55% PVE, respectively. These two QTLs were also detected as major QTLs under the unconditional QTL mapping. Of the nine QTLs detected in both strategies, five conditional QTLs changed additive effects significantly and the other four showed similar effects to the unconditional ones. Two extra conditional QTLs identified for LLS and seven QTLs were detected only under unconditional situation.

A total of 13 conditional QTLs of LLS|TNB were detected. Six conditional QTLs were also detected by unconditional mapping. Among them, two QTLs (*qLLSA1-3* and *qLLSB6-6*) were detected in multiple environments, but none of them were detected with PVE more than 10%. Of the six QTLs detected in both strategies, five conditional QTLs changed additive effects obviously and one conditional QTL showed similar additive effect to their corresponding unconditional QTLs. Conditioned on TNB, however, 10 unconditional QTLs were not identified again, but seven extra QTLs were detected.

## Discussion

QTL analysis has been well documented to dissect the genetic basis of important, complex traits in cultivated peanut [43–49]. However, due to the nature of low genetic diversity and polyploidy in peanut, precisely locating the QTLs controlling the traits of interest is still challenging. In this study, we conducted QTL mapping using our previously constructed genetic map with the highest number of markers (1,685 marker loci) among so far published population-specific linkage maps for tetraploid peanut [29]. Totally, we detected 20 QTLs for LLS and 32 QTLs for three plant-type-related traits. The number of QTLs was much more than the published QTL mapping in a single population of peanut [26,44], suggesting the detection power of QTL mapping was significantly improved.

QTL analysis in single environment is limited in predicting QTL positions and the stability of QTLs as well as the magnitude of genetic effects on target traits [50,51]. In addition, QTL that has obvious genetic effect could be preferentially identified in different environments. In this study, we performed QTL analysis based on phenotypic data from multiple environments. Although most of detected loci were environment-specific, six QTLs were identified in multiple environments and hence were stable in phenotypic expression. For example, a QTL for LLS resistance, namely *qLLSB6-2*, flanked by Ahsnp539 and Ahsnp136, was expressed with 11.66% PVE in NC12 and with 12.9% PVE in NC11. And a QTL for TBN, namely *qTNBB6-3*, flanked by AHGS1431 and Ahsnp690, was expressed with 8.1% PVE in NC13 and with 13.44% PVE in WH13. Due to the limitation of experimental material in the present study, the identified major QTLs and the markers linked the studied traits are needed further validation before they are deployed in MAS.

For LLS resistance, we identified 20 QTLs. Of which, six QTLs showed PVE over 10%, and other QTLs were basically significant loci with relatively lower phenotypic contributions. These results indicated that the resistance to LLS in cultivated peanut was controlled by several major QTLs and many minor QTLs, consistent with previous research results [15,46]. When comparing the QTLs for LLS in this study with previous studies (S3 Table) [14,15,46,52,53,54], six major and 10 minor QTLs for LLS in our study were shown to be novel QTLs. This could be due to different genetic backgrounds of the used materials. We also found that the physical region of *qLLSA1-2* was involved in a previously identified QTL *qF2LS3* [46], and *qLLSA4-1* was involved in QTL_LLS_09 which reported by Khedikar et al. (2010) [14]. The physical region of *qLLSB6-3* was overlapped with QTL_R5-LLS_13 in other study [15], *qLLSB10* overlapped with reported QTLs of QTL_LLS_11 and *qF2LS2* [14,46]. Therefore, these four QTLs were repeatedly detected in different populations. With respect to the QTLs for plant-type-related traits, no similar segment was found between our study and previously studies [26,27,55,56].

Nucleotide-binding—leucine-rich repeat (NB-LRR)-encoding genes usually confer resistance against pests and diseases. Bertioli et al. (2016) [57] identified 345 and 397 of these genes in the *A*. *duranensis* and *A*. *ipaensis* genotypes, which serve as proxies for the A and B subgenomes of peanut, respectively. We therefore compared the physical locations of QTLs for LLS resistance in our study with the NB-LRR—encoding genes and found five QTLs for LLS resistance were associated with these genes (S4 Table). A major QTL *qLLSB6-7* for resistance to LLS identified on B6 of ICGV 86699 [31] resides in a cluster of six NB-LRR—encoding genes covering 3.9 Mb. Another QTL *qLLSB1* was identified on a segment that covering 8.9 Mb of chromosome B1 which contains five NB-LRR—encoding genes. The genes harbored on these genome segments provide good disease resistance and warrant further investigation.

In the RIL population of Zhonghua 5 × ICGV 86699, the LLS resistance alleles at 20 QTL regions were contributed by ICGV 86699. This level of resistance to LLS has never been identified in “pure” cultivated peanut and in order to widen primary genetic pool, wild species were used [58–62]. From the pedigree of ICGV 86699, we knew that the resistance to LLS was introgressed from wild species of *A*. *duranensis* and *A*. *batizocoi* [31]. Due to that genome fragments from wild peanut were transferred into cultivated peanuts, this population therefore had high genetic variation and the map had so many polymorphic markers [29]. In the previous studies of QTL mapping for resistance to LLS, GPBD 4 was usually used as a resistant parent [14,15,53]. The source of resistance of GPBD 4 can be traced back to wild species of *A*. *cardenasii* [63], which is different from this study.

QTL cluster is an important central concept in genetical genomics [64] and associative traits tend to share regions with QTLs [65–67]. Several studies in plants have mapped QTLs for disease resistances which overlap with those for plant-type-related traits [20,21,68,69]. In an overview of QTL distribution in this study, 10 QTLs including eight major QTLs were clustered in five genetic regions on chromosomes A5, A9 and B6. The genomic regions of QTL cluster IV and V harbored co-localized QTLs for LLS and TNB while cluster I harbored co-localized QTL for LLS and HMS. These results provided evidence that correlation of LLS and plant-type-related traits could be genetically constrained. In peanut, Shoba et al. (2013) [50] found that markers PM 384, pPGPseq5D5, PM 3, PMc 588 and PM 343 associated with number of branches and LLS severity score through single marker analysis, also suggesting genetic correlation existed between LLS and plant type. The genetic regions with clustered robust QTLs of this study are worth of further investigation due to the importance of genetic control of LLS as well as plant type.

The conditional analysis provides an efficient method for dissecting the genetic interrelationship between traits at individual QTL level [9]. Comparing the QTLs of LLS analyzed by unconditional and conditional mappings, the following four possible implications existed in this study: (1) the QTLs, such as *qLLSB6-2* and *qLLSA5*, were detected by unconditional mapping and not be recaptured when LLS was conditioned on HMS, LLB or TNB, suggesting that these QTLs for LLS were completely depended on the plant-type-related traits; (2) the loci, such as *qLLSA2* and *qLLSB2-1*, were identified by conditional, but not unconditional mapping, indicating that such QTLs were completely suppressed by conditional plant-type-related traits; (3) the QTLs, such as *qLLSA6-3*, identified by two mapping methods had similar effects, indicating that the QTLs for LLS were independent of conditional traits; (4) the QTLs, such as *qLLSB6-4* and *qLLSB6-6*, identified by both unconditional and conditional mapping methods showed significantly changed effects, indicating that these QTLs for LLS were partially influenced by plant-type-related traits. Of the 16 unconditional QTLs for LLS, seven, seven and 10 QTLs were entirely determined by HMS, LLB and TNB respectively; five QTLs each were partial contributions from HMS, LLB and TNB, respectively; four, four and one QTLs were independent of HMS, LLB and TNB, respectively. These results suggested that TNB contributed the strongest influence on LLS.

This study improved our understanding of genetic basis of LLS and provided several QTLs for LLS, HMS, LLB and TNB. From the QTL cluster and conditional analysis, we found that interrelationship existed between LLS and plant-type-related traits. However, challenging questions remain such as how to fast-expanding the population to meet the needs of fine-mapping in peanut. Further validation of major QTLs and markers lined the studied traits will be proceeded before applied in MAS. Besides, support from other omics researches, like transcriptomics, should also be considered to promote genetic analysis and the isolation of favorable alleles based on this study.

## Supporting Information

S1 Fig The schematic diagram of HMS and LLB investigated in this study. HMS and LLB were measured in the unit of centimeter (cm). HMS: height of main stem; LLB: length of the longest branch.
(EPS)

S2 Fig Frequency distribution of RIL populations for LLS, HMS, LLB and TNB traits in multi-environments. (EPS)

S3 Fig Distribution of identified QTLs for LLS, HMS, LLB and TNB on genetic linkage maps. Scale bars on the left side describe map distance in centimorgans. Markers were shown on left side of the linkage groups. The QTLs for LLS, HMS, LLB and TNB were shown by boxes filled with red, green, blue and purple, respectively. The regions for QTL clusters were designed as yellow box on chromosome bars. Each cluster includes at least one major QTL. LLS: late leaf spot disease; HMS: height of main stem; LLB: length of the longest branch; TNB: total number of branches.
(PDF)

S1 Table The marker sequences of the linkage map used for QTL mapping. (XLSX)

S2 Table QTLs of LLS and plant-type-related traits identified only in joint analysis. (XLSX)

S3 Table The identified QTLs (or nearest marker) for LLS (or LS) of peanut in previous and this studies. (XLSX)

S4 Table **(A) Information of QTLs for LLS resistance in this study in which genomic regions harbored NB-LRR—encoding genes.** Physical locations of each marker on the genome were determined by searching SNP markers via blast and SSR markers using ePCR to align to AA and BB genome sequence. **(B) Genes with NBS domains in the QTL covering segments of pseudomolecule.**
(XLSX)
