# Supplementary material for: Quantitative Trait Locus Analysis of Late Leaf Spot Resistance and Plant-Type-Related Traits in Cultivated Peanut (Arachis hypogaea L.) under Multi-Environments
Source: PLoS One. 2016 Nov 21;11(11):e0166873. doi: 10.1371/journal.pone.0166873 (PMC5117734; doi:10.1371/journal.pone.0166873)
Supplement: S3 Fig — Scale bars on the left side describe map distance in centimorgans. Markers were shown on left side of the linkage groups. The QTLs for LLS, HMS, LLB and TNB were shown by boxes filled with red, green, blue and purple, respectively. The regions for QTL clusters were designed as yellow box on chromosome bars. Each cluster includes at least one major QTL. LLS: late leaf spot disease; HMS: height of main stem; LLB: length of the longest branch; TNB: total number of branches. (PDF) [file pone.0166873.s003.pdf]

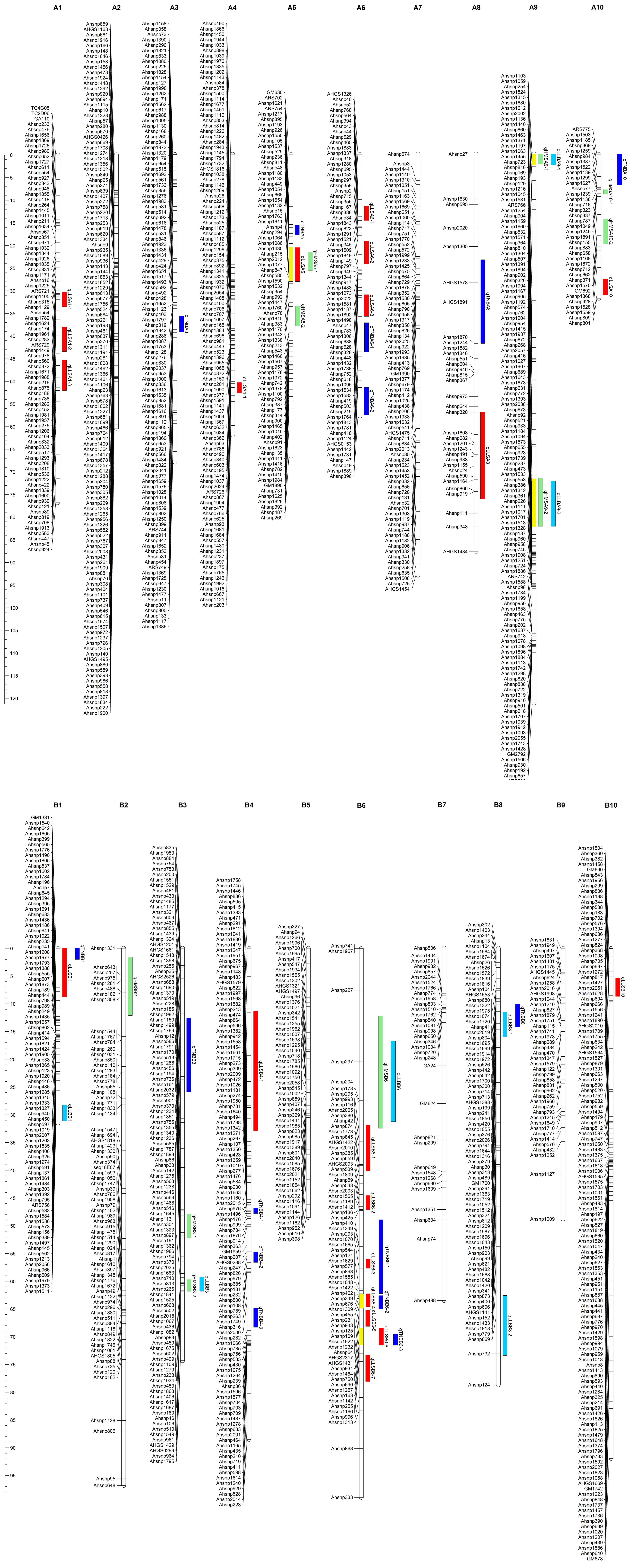
A1
A2
A3
A4
A5
A6
A7
A8
A9
A10
B1
B2
B3
B4
B5
B6
B7
B8
B9
B10
